# Insights into the ligand binding specificity of SREC‐II (scavenger receptor expressed by endothelial cells)

**DOI:** 10.1002/2211-5463.13260

**Published:** 2021-09-12

**Authors:** Catherine Wicker‐Planquart, Pascale Tacnet‐Delorme, Laurence Preisser, Samy Dufour, Yves Delneste, Dominique Housset, Philippe Frachet, Nicole M Thielens

**Affiliations:** ^1^ CNRS CEA IBS University of Grenoble Alpes Grenoble France; ^2^ CHU Angers Inserm CRCINA, SFR ICAT Univ Angers Université de Nantes Angers France

**Keywords:** complement C1q, interaction, scavenger receptor SREC‐II, maleylated BSA, surface plasmon resonance, stably transfected cells

## Abstract

SREC‐II (scavenger receptor expressed by endothelial cells II) is a membrane protein encoded by the *SCARF2* gene, with high homology to class F scavenger receptor SR‐F1, but no known scavenging function. We produced the extracellular domain of SREC‐II in a recombinant form and investigated its capacity to interact with common scavenger receptor ligands, including acetylated low‐density lipoprotein (AcLDL) and maleylated or acetylated BSA (MalBSA or AcBSA). Whereas no binding was observed for AcLDL, SREC‐II ectodomain interacted strongly with MalBSA and bound with high affinity to AcBSA, a property shared with the SR‐F1 ectodomain. SREC‐II ectodomain also interacted with two SR‐F1‐specific ligands, complement C1q and calreticulin, with affinities in the 100 nm range. We proceeded to generate a stable CHO cell line overexpressing full‐length SREC‐II; binding of MalBSA to these cells was significantly increased compared with nontransfected CHO cells. In contrast, no increase in binding could be detected for C1q and calreticulin. We show for the first time that SREC‐II has the capacity to interact with the common scavenger receptor ligand MalBSA. In addition, our data highlight similarities and differences in the ligand binding properties of SREC‐II in soluble form and at the cell surface, and show that endogenous protein ligands of the ectodomain of SREC‐II, such as C1q and calreticulin, are shared with the corresponding domain of SR‐F1.

## Graphical abstract

We investigated the ligand binding properties of SREC‐II, a receptor with high homology to scavenger receptor SR‐F1. Both SREC‐II expressed at the surface of stably transfected CHO cells and soluble recombinant SREC‐II ectodomain bind to the emblematic scavenger receptor ligand maleylated BSA, but not to acetylated LDL. Only soluble SREC‐II interacts with C1q and calreticulin, two endogenous protein ligands of SR‐F1, which rises new hypotheses about SREC‐II possible function.

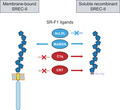

**Abbreviations**
AcBSA: acetylated BSA
AcLDL: acetylated LDL
CRT: calreticulin
EGF: epidermal growth factor
LDL: low‐density lipoprotein
MalBSA: maleylated BSA
SPR: surface plasmon resonance
SREC: scavenger receptor expressed by endothelial cells
SR‐F: scavenger receptor class F
TSA: thermal shift assay

The scavenger receptor expressed by endothelial cells SREC‐II, a membrane protein encoded by the *SCARF2* gene, was discovered by identification of paralogous sequences with *SCARF1*, the gene coding for scavenger receptor expressed by endothelial cells SREC‐I [1]. SREC‐II has significant amino acid similarity to SREC‐I, and both receptors showed a similar transcriptional expression profile across a range of human tissues, including heart, lung, ovary and spleen [1, 2]. Although SREC‐I was initially cloned from cultured umbilical vein endothelial cells, its expression has since been reported in many cell types, including sinusoidal endothelial cells, epithelial cells, dendritic cells and macrophages (reviewed in [3]). Studies exploring SREC‐II cellular expression at the protein level are lacking. On a functional point of view, SREC‐II differs from SR‐F1 (SREC‐I) [4] as it displays no known scavenger activity and is unable to recognize and endocytose modified low‐density lipoproteins (LDL) such as acetylated LDL (AcLDL) and oxidized LDL [1]. This lack of scavenging activity explains why SREC‐II then retained its name, whereas SREC‐I was named SR‐F1 (for scavenger receptor type F) in the new nomenclature proposed by PrabhuDas *et al*. in 2017 [5].

SREC‐II is a transmembrane protein that contains ten epidermal growth factor (EGF)‐like repeats in the extracellular domain and shares 52% sequence identity with SR‐F1 over the ectodomain of mature protein (402 amino acids). This suggests that the ectodomains of both receptors presumably share some biological properties. By contrast, SREC‐II long cytoplasmic tail (403 amino acids), rich in positively charged residues such as arginine (11.5%) and lysine (5.9%), has only 22% sequence identity with SR‐F1 intracellular domain, and dissimilar potential phosphorylation sites [1], which suggests that different proteins may interact with the intracellular domain of both receptors, resulting in different signalling transduction.

The only interaction described so far for SREC‐II, obtained with the murine protein, occurs *via* its ectodomain that binds to SR‐F1 corresponding domain and evokes cell aggregation [1]. The SR‐F1/SREC‐II heterodimerization also suppresses SR‐F1 ability to bind modified LDL [1]. No other interaction with SREC‐II ectodomain has yet been reported. We have recently reinvestigated SR‐F1 interactions with endogenous proteins complement C1q and the multifunctional calcium‐binding chaperone calreticulin (CRT), which likely contribute to its role in the elimination of apoptotic cells, a process called efferocytosis [6]. Although apoptotic cell sensing was reported to be specific of SR‐F1 [7], we were interested in exploring SREC‐II possible interactions with C1q and CRT, and with an emblematic scavenger receptor ligand, maleylated BSA (MalBSA). For this purpose, we produced the extracellular domain of SREC‐II in a recombinant form and generated stably transfected CHO cells to study these interactions at the molecular level and at the cell surface. Surprisingly, the SREC‐II soluble ectodomain and the cell surface‐anchored SREC‐II entity do not exhibit the same binding properties, rising new hypotheses about its possible biological function.

## Materials and methods

### Proteins and reagents

Purified human C1q and C1q‐derived collagen‐like regions (CLR) and globular regions (GR) were prepared as described previously [8]. Recombinant human CRT and SR‐F1 ectodomain (aa 20‐421) were produced according to published procedures [6, 9]. MW and A_1%,1__cm_ at 280 nm used for protein quantification were 459 300 g·mol^−1^ and 6.8 for C1q, 189 900 g·mol^−1^ and 2.1 for C1q‐CLR, 48 000 g·mol^−1^ and 9.3 for C1q‐GR, 49 431 g·mol^−1^ and 16.5 for CRT, and 49 000 g·mol^−1^ and 16.7 for SR‐F1(20‐421). N‐glycosidase F (PNGase F) was purified from cultures of *Flavobacterium meningosepticum* as described by Aude *et al*. [10]. LDL (Sigma‐Aldrich, St‐Quentin‐Fallavier, France) and AcLDL (Acris Antibodies, Hereford, Germany) concentrations were determined using the Quick Start Bradford 1x Dye Reagent (Bio‐Rad, Marnes‐la‐Coquette, France). The protein contents represent approximately a quarter of the total weight of the LDL samples. BSA and acetylated BSA (AcBSA) were purchased from Sigma‐Aldrich. BSA maleylation was performed essentially as described by Butler and Hartley [11]. Briefly, maleic anhydride (800 mg at 40 mg·mL^−1^) was added slowly to ice‐cooled BSA (200 mg BSA at 10 mg·mL^−1^) in 50 mm Tris/HCl, pH 8.8, while the solution pH was maintained between 8.5 and 9 by the addition of sodium carbonate (about 2.28 g). The reaction was considered to be complete when the pH remained constant. The maleylated protein was concentrated to 5 mL over an Amicon Ultra Centrifugal Filter (Merck Chimie, Fontenay‐sous‐Bois, France) and passed through 2 PD‐10 desalting columns (Cytiva, Velizy‐Villacoublay, France) equilibrated in H_2_O, pH 8.8. The concentration of MalBSA was 30 mg·mL^−1^ (Bradford determination), and the extent of maleylation, calculated from absorbance measurement at 250 nm, was 47 maleylated lysines out of 59 in native BSA. Alexa Fluor 568‐succinimidyl ester and Alexa Fluor 488 C5‐maleimide were from Thermo Fisher Scientific (Carlsbad, CA, USA). Recombinant human CRT labelling with the succinimidyl ester conjugate and maleylated BSA labelling with the C5‐maleimide were performed according to the manufacturer’s protocol.

### Construction of expression plasmids for SREC‐II

The oligonucleotides used (Eurogentec, Angers, France) are described in Supporting Information.

A synthetic DNA coding for the extracellular moiety of SREC‐II (amino acids 1‐442) was purchased from GeneCust (Dudelange, Luxemburg) (Supporting Information). A SREC‐II DNA fragment containing an EcoRI restriction site at the 5’‐end and a PacI restriction site at the 3’‐end was generated by PCR using Pfu polymerase (Agilent Technologies, Les Ulis, France), the synthesized DNA fragment, SREC‐II‐EcoRIF and SREC‐II‐PacR oligonucleotides. The pcDNA3.1‐SREC‐II(1‐442) plasmid was obtained by inserting the PCR fragment in the pcDNA3.1‐SR‐F1 plasmid already described [6] in place of SR‐F1 ectodomain sequence using EcoRI and PacI restriction sites. The recombinant protein ends at amino acid 442 (Gly) of the SREC‐II sequence followed by 3 amino acids (Leu‐Ile‐Lys) and 8 His residues. The sequence was verified by DNA sequencing (Eurofins Genomics, Köln, Germany).

### Production of SREC‐II ectodomain in 293‐F cells and protein purification

Transfection of 293‐F cells with the pcDNA3.1‐SREC‐II(1‐442) plasmid, generation of stably transfected cells and purification of the His‐tagged recombinant protein from the cell culture supernatant were achieved as described [6]. The only modifications were in Ni‐NTA resin (Qiagen, Courtaboeuf, France) washing buffer (Tris‐HCl 50 mm, pH 7.4, 0.15 m NaCl (TBS), containing 50 mm imidazole) and elution buffer (150 mm imidazole in TBS) compositions, where NaCl concentrations were lowered. The purified protein was dialysed against TBS and concentrated to 0.2‐1 mg·mL^−1^ by ultrafiltration on an Amicon Ultra‐4 Centrifugal Filter (10 kDa cut‐off).

The molar concentration of SREC‐II (amino acids 44‐ 442)was quantified using an absorption coefficient A_1%,_ 1 cm at 280 nm of 14.8 (https://web.expasy.org/protparam/) and a molecular mass value of 55.8 kDa, as determined by mass spectrometry (average of 3 independent measurements).

### Biochemical and biophysical characterization

Protein analysis by sodium dodecyl sulfate/polyacrylamide gel electrophoresis (SDS/PAGE), N‐terminal sequence and mass spectrometry analyses, recording of circular dichroism (CD) spectra, thermal shift assay (TSA) experiments and PNGase F treatment were performed as described by Wicker‐Planquart *et al*. [6]. Prior to N‐terminal sequence determination, removal of the N‐terminally blocked group of SREC‐II was achieved by incubation of the protein for 6 h at 50 °C with Pfu pyroglutamate aminopeptidase (Takara Bio, Shiga, Japan) (2 mU·nmol^−1^ protein) in the presence of 1 mm EDTA and 10 mm dithiothreitol.

### Surface plasmon resonance (SPR) analyses and data evaluation

All interaction experiments were carried out at 25 °C on a Biacore 3000 or a T200 instrument (Cytiva). Protein ligands were covalently linked to CM5 sensor chips (Cytiva) in HBS (0.01 m HEPES pH 7.4, 0.15 m NaCl) containing either 0.005% (Biacore 3000) or 0.05% (T200) surfactant P20 using amine coupling via reactive esters according to the manufacturer’s instructions (Cytiva). Ligand dilution conditions and immobilization levels were as follows: AcBSA, 25 µg·mL^−1^ in 10 mm formate, pH 3 (480‐900 RU); MalBSA, 30 µg·mL^−1^ in 10 mm formate, pH 3 (78 RU); BSA, 5‐25 µg·mL^−1^ in 10 mm sodium acetate, pH 4 (370‐3500 RU); SR‐F1(20‐421), 7‐14 µg·mL^−1^ in 10 mm sodium acetate, pH 5 (2900–5200 RU); SREC‐II(44‐442), 7.5‐12.5 µg·mL^−1^ in 10 mm sodium acetate, pH 5 (2000–5200 RU); and C1q, 35 µg·mL^−1^ in 10 mm sodium acetate, pH 5.5 (16 000–18 300 RU).

A flow cell submitted to the coupling steps without protein immobilization was used as a reference for SR‐F1, SREC‐II and CRT. Immobilized BSA was used as the reference surface for AcBSA, MalBSA and C1q. The specific binding signals were obtained by subtracting the signals over the reference surface.

Binding was measured at a flow rate of 20 μL·min^−1^ (30 μL·min^−1^ for CRT) in TBS, pH 7.4, containing 0.005% (Biacore 3000) or 0.05% (T200) surfactant P20 and 2mM CaCl_2_ (TBS‐Ca‐P). LDL and AcLDL interactions were recorded in the absence of surfactant. The specific binding signal was obtained by subtracting the signal from the reference surface. Surfaces were regenerated by 30‐s injections of 1 m NaCl and 10 mm EDTA.

Determination of kinetic parameters was performed by global fitting of both the association and dissociation phases for at least five concentrations, either to a 1:1 Langmuir binding model or to a two‐state reaction (conformational change) binding model, using the BIA evaluation 3.2 (Biacore 3000) or biacore T200 evaluation 2.0 software (Cytiva). Buffer blanks were subtracted from the data sets. The apparent equilibrium dissociation constants (*K*_D_) were calculated from the rate constants as the *k*_d_/*k*_a_ ratio for the 1 : 1 binding model and using the following formula: *K*_D_ = 1/[(*k*_a1_/*k*_d1_) (1 + *k*_a2_/*k*_d2_)] for the two‐state reaction model. Chi2 values were below 4 in all cases.

### Generation of SREC‐II‐expressing CHO cells

The cDNA encoding the scavenger receptor SREC‐II (OriGene Technologies, Rockville, MD, USA) was subcloned in the expression vector pcDNA3.1 (Thermo Fisher Scientific, Invitrogen, Carlsbad, CA, USA). CHO cells (ATCC, Manassas, VA, USA) were transfected using Lipofectamine 2000 (Thermo Fisher Scientific) and cultured in HAM’s F12 medium supplemented with 5% fetal bovine serum and selected with blasticidin (all from Thermo Fisher Scientific). SREC‐II‐expressing CHO cells were sorted by flow cytometry (FACSAria II; BD Biosciences, San Jose, CA, USA) using anti‐SREC‐II mAb (R&D Systems, Minneapolis, MN, USA). Bound antibodies were revealed using FITC‐labelled goat anti‐mouse IgG Ab (BD Biosciences). The expression of SREC‐II was verified by flow cytometry before use in binding assays. Isotype control mAb was from R&D Systems.

### Flow cytometric analysis for C1q, MalBSA and CRT binding

Cells were incubated with C1q (final concentration 80 µg·mL^−1^ in PBS containing 3% (w/v) BSA) on ice for 40 min and then incubated for 45 min with mouse anti‐C1q Abs (A201; Quidel, San Diego, CA, USA) (1 mg·mL^−1^) diluted 1/100 followed by a Cy‐3‐labelled anti‐mouse antibodies (Jackson ImmunoResearch, Ozyme, Saint‐Cyr‐L’Ecole, France). Alexa Fluor 488‐labelled MalBSA or Alexa Fluor 568‐labelled recombinant human CRT at 1, 5 and 10 µg·mL^−1^ in PBS containing 3% (w/v) BSA were incubated with cells for 30 min on ice. Cells were then washed with PBS and immediately analysed by flow cytometry.

FACS analyses were performed on a MACSQuant VYB Cytometer (Miltenyi Biotec, Paris, France) and collected data were analysed with MACSQuant software. Result was expressed as MFI (median fluorescence intensity) ratio (MFI of the labelled sample/MFI of the control).

### Statistical analysis

Two‐tailed *P* value was obtained using *t*‐test for paired samples after validation by a Shapiro–Wilk normality test (graphpad Prism 8.0).

## Results

### Production and biochemical characterization of SREC‐II ectodomain

**Fig. 1:**
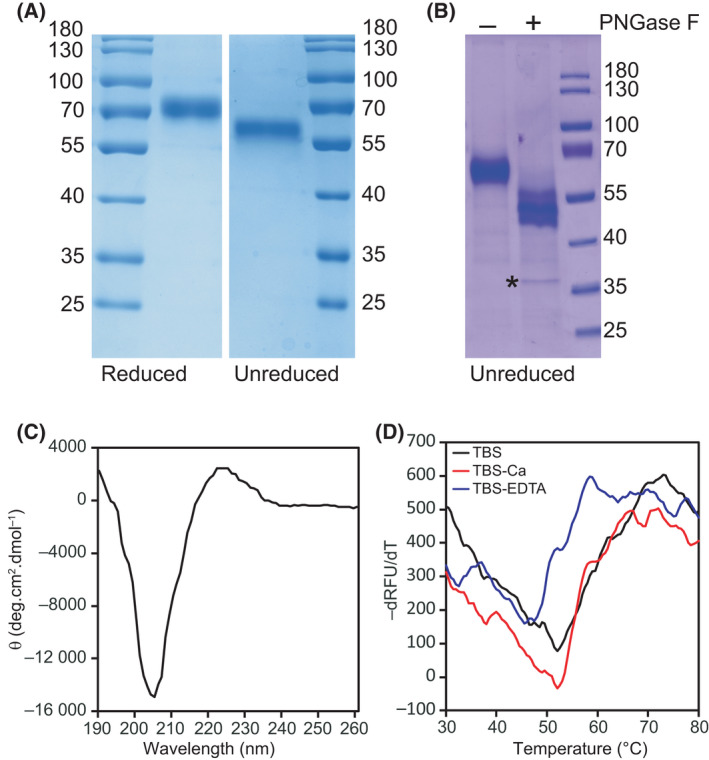
Analysis of SREC‐II ectodomain produced in mammalian cells. (A) SREC‐II(44‐442) was analysed by SDS/PAGE under reduced or unreduced conditions. Two µg was loaded on the gel. The molecular mass values of the marker are indicated in kDa. (B) PNGase F deglycosylation of SREC‐II. Digestion products were analysed by SDS/PAGE (unreduced conditions). PNGase F (35 kDa) is indicated by a star. The molecular masses (kDa) of the molecular weight marker are indicated. (C) CD spectrum of SREC‐II(44‐442). The protein was used at a concentration of 8 µm in 10 mm sodium phosphate and 150 mm NaF (pH 8). Ten spectra were acquired at a scan rate of 20 nm·min^−1^ in the far‐UV region (200–260 nm). (D) Thermal shift analysis of SREC‐II (44‐442) in TBS (black line), in the presence of 2 mm CaCl_2_ (red line) or 2 mm EDTA (blue line). Two µg of protein was used per well.

SREC‐II(44‐442), corresponding to the ectodomain of the receptor fused to a C‐terminal His‐tag, was produced in 293‐F cells and purified from the cell culture supernatant by nickel affinity chromatography. A mean amount of 80 µg SREC‐II(44‐442) was purified from 100 mL cell culture. SDS/PAGE analysis showed that the protein migrates as a broad band, probably due to heterogeneous glycosylation (Fig. 1A). Migration of unreduced versus reduced protein was notably different, due to the high number of predicted cysteines (36) in SREC‐II protein. The molecular mass, as determined by MALDI‐TOF mass spectrometry, was 55.8 ± 0.3 kDa (mean ± SE of 3 independent measurements), accounting for the polypeptide chain (44.5 kDa) plus an extra mass of 11.3 kDa, which is due to the glycosylated state of the protein. Five N‐glycosylation sites are indeed predicted in SREC‐II protein (N83, N218, N310, N365 and N413). SREC‐II glycosylation was further confirmed by PNGase F digestion of the protein (resulting in the cleavage of Asn‐linked oligosaccharides) followed by SDS/PAGE analysis, where a substantial shift in SREC‐II migration was observed (Fig. 1B). Edman degradation of SREC‐II ectodomain yielded no N‐terminal sequence, likely due to cyclization of the N‐terminal glutamine residue. Pretreatment of the protein with pyroglutamate aminopeptidase yielded the Glu‐Leu‐Asn‐Pro‐Arg sequence, in accordance with SREC‐II sequence from residue 45, confirming conversion of N‐terminal glutamine 44 to pyroglutamate.

SREC‐II(44‐442) correct folding was assessed by circular dichroism (CD) analysis between 190 and 260 nm (Fig. 1C). The far‐UV CD spectrum of SREC‐II was characteristic of EGF‐containing proteins, with a minimum negative signal at 205 nm and a slightly positive maximum signal at 224 nm, due to the presence of tryptophan and tyrosine residues in recombinant EGF modules [12]. CD spectra of SREC‐II and SR‐F1 [6] were similar. SREC‐II(44‐442) stability was also analysed by thermal shift assay (TSA). A melting temperature (T_m_) of 52 ± 2 °C was measured in TBS (Fig. 1D), a value higher than that recorded for SR‐F1 (46 ± 1 °C, [6]). Unlike SR‐F1, increasing NaCl concentrations in TBS destabilized SREC‐II protein, with a drop of up to 7 °C in the T_m_ value (Table 1). Addition of 2 mm CaCl_2_ restored the initial *T*_m_, thus stabilizing the protein, whereas the presence of 2 mm EDTA lowered the *T*_m_ value to about 45 °C (Table 1), contrasting with the lack of influence of CaCl_2_ or EDTA observed previously for SR‐F1 [6]. These results led us to adapt SREC‐II purification, especially regarding the composition of binding, washing and elution buffers used during the nickel affinity chromatography, where NaCl final concentration was dropped from 0.3 m (used for SR‐F1 ectodomain) to 0.15 m. All interaction experiments were performed in the presence of 2 mm CaCl_2_.

**Table 1:** Influence of calcium ions and NaCl concentration on SREC‐II stability, measured by thermal shift assay analysis. Values are the means ± SE of two to three separate experiments.

[NaCl] (m)	*T*_m_ ( °C)		
	TBS	TBS + 2 mm CaCl_2_	TBS + 2 mm EDTA
0.15	51.8 ± 0.3	52.8 ± 0.6	45.3 ± 0.3
0.3	47.2 ± 0.3	53.8 ± 1.5	
0.42	44.5 ± 0.4	53.5 ± 0.8	

### Molecular interaction analyses

#### Interaction of SREC‐II ectodomain with scavenger receptor ligands such as modified LDL or BSA

**Fig. 2:**
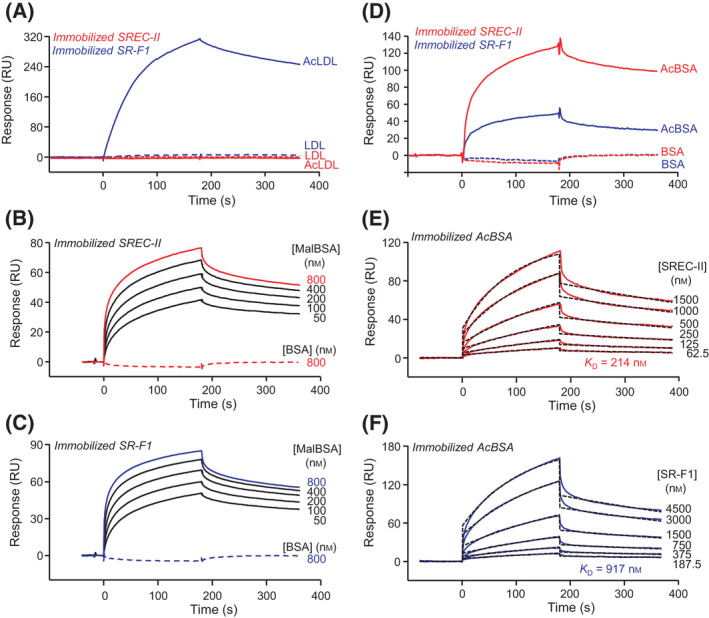
SPR analyses of SREC‐II ectodomain interactions with modified LDL and BSA. (A) AcLDL and LDL (0.1 µg·mL^−1^) were injected over immobilized SREC‐II(44‐442) (3400 RU). Immobilized SR‐F1(20‐421) (2900 RU) was used as a control. (B, C) Serially diluted MalBSA (50‐800 nm) was injected over (B) immobilized SREC‐II(44‐442) (5250 RU) or (C) immobilized SR‐F1(20‐421) (4260 RU). (D) AcBSA and BSA (1 µm) were injected over immobilized SREC‐II(44‐442) (3400 RU) and SR‐F1(20‐421) (2900 RU). (E) SREC‐II(44‐442) and (F) SR‐F1(20‐421) were serially diluted and injected over immobilized AcBSA (900 RU). Fits (shown as dotted lines) and apparent *K*_D_ values were obtained by global fitting of the data using a 1:1 Langmuir binding model. The binding experiments were performed in TBS‐Ca (A) and TBS‐Ca‐P (B‐F) at a flow rate of 20 µL·min^−1^. The data shown are representative of at least 2 separate experiments using the T200 or the Biacore 3000 apparatus.

Acetylated LDL (AcLDL) is a classical ligand of scavenger receptors, including SR‐F1 [6, 13]. Surface plasmon resonance (SPR) was used to investigate acetylated and unmodified LDL binding to SREC‐II(44‐442), using SR‐F1(20‐421) as a control. Preliminary experiments were performed with AcLDL immobilized on the sensor chip in the presence of surfactant P20 and soluble SREC‐II and SR‐F1 injected over the surface in the absence of P20. Under these conditions, binding of both proteins to the AcLDL surface was observed. Since P20 detergent may have altered the lipidic part of AcLDL, we also tested the reverse configuration, that is soluble AcLDL injected in the absence of detergent (to preserve its integrity) over the immobilized receptors. In this configuration, SREC‐II(44‐442) did not bind intact AcLDL, as already reported by Ishi *et al*. [1], as opposed to SR‐F1 ectodomain (Fig. 2A). Unmodified LDL did not interact with either SREC‐II or SR‐F1 ectodomains (Fig. 2A).

Apart from AcLDL, MalBSA has been reported as another common ligand of scavenger receptors [14, 15]. We therefore tested binding of MalBSA to immobilized SREC‐II(44‐442) and SR‐F1(20‐421). Both ectodomains bound MalBSA in a dose‐dependent manner (Fig. 2B,C). However, the binding curves could not be fitted to usual models (1:1 Langmuir binding model or two‐state reaction model) and we were thus unable to calculate an apparent *K*_D_ value. Using the reverse configuration, binding of soluble SREC‐II and SR‐F1 could also be observed (not shown). However, covalent immobilization of MalBSA was not very efficient, likely due to its high number of negative charges. In addition, regeneration of the surface could not be achieved using common regeneration solutions including NaCl 1–2 m, Na acetate 1–2 m, MgCl_2_ 1–3 m and NaOH 5‐25 mm, which precluded kinetic analysis of the interactions. Under the same conditions, SREC‐II and SR‐F1 did not interact with unmodified BSA (Fig. 2B,C).

We also investigated the interaction of SREC‐II with another modified BSA molecule, that is, acetylated BSA (AcBSA) that bound dose‐dependently to immobilized SREC‐II (Fig. 2D). Using the reverse configuration (immobilized AcBSA and soluble SREC‐II or SR‐F1; Fig. 2E,F), we were able to determine apparent *K*_D_ values of 2.0 ± 0.2 10^−7^ m and 8 ± 1 10^−7^ m for SREC‐II and SR‐F1, respectively. Under these conditions, SREC‐II bound thus AcBSA with a higher affinity than SR‐F1, the difference arising from a higher association rate constant (Table 2).

**Table 2:** Kinetic and dissociation constants for SREC‐II interaction with C1q or CRT and of SREC‐II or SR‐F1 with AcBSA. Values are the means ± SE of two to three separate experiments.

Soluble interactant	Constants	Immobilized interactant		
		C1q	AcBSA	SREC‐II
SREC‐II	*k*_a_ (m ^−1^ s^−1^)	2.0 ± 0.6 × 10^4^	7.8 ± 1.2 × 10^3^	
	*k*_d_ (s^−1^)	1.0 ± 0.5 × 10^−3^	1.5 ± 0.1 × 10^−4^	
	*K*_D_ (m)	4.5 ± 1.2 × 10^−8^	2.0 ± 0.2 × 10^−7^	
CRT	*k_a1_ (m ^−1^ s^−1^)			6.3 ± 3.1 × 10^3^
	*k_a2_ (s^−1^)			2.3 ± 0.3 × 10^−3^
	*k_d1_ (s^−1^)			9.2 ± 0.4 × 10^−3^
	*k_d2_ (s^−1^)			4.4 ± 0.1 × 10^−4^
	*K_D_ (m)			2.9 ± 0.9 × 10^−7^
SR‐F1	*k*_a_ (m ^−1^ s^−1^)		2.0 ± 0.3 × 10^3^	
	*k*_d_ (s^−1^)		1.5 ± 0.1 × 10^−3^	
	*K*_D_ (m)		7.9 ± 1.0 × 10^−7^	

* The association (*k*_a1_, *k*_a2_) and dissociation (*k*_d1_, *k*_d2_) rate constants of the C1q/CRT interaction were determined by global fitting of the data using a two‐state reaction binding model. The resulting dissociation constant *K*_D_ was determined from the (*k*_d1_/*k*_a1_)(*k*_d2_/*k*_a2_) ratios.

#### Interaction of SREC‐II ectodomain with endogenous proteins such as complement C1q and calreticulin (CRT)

**Fig. 3:**
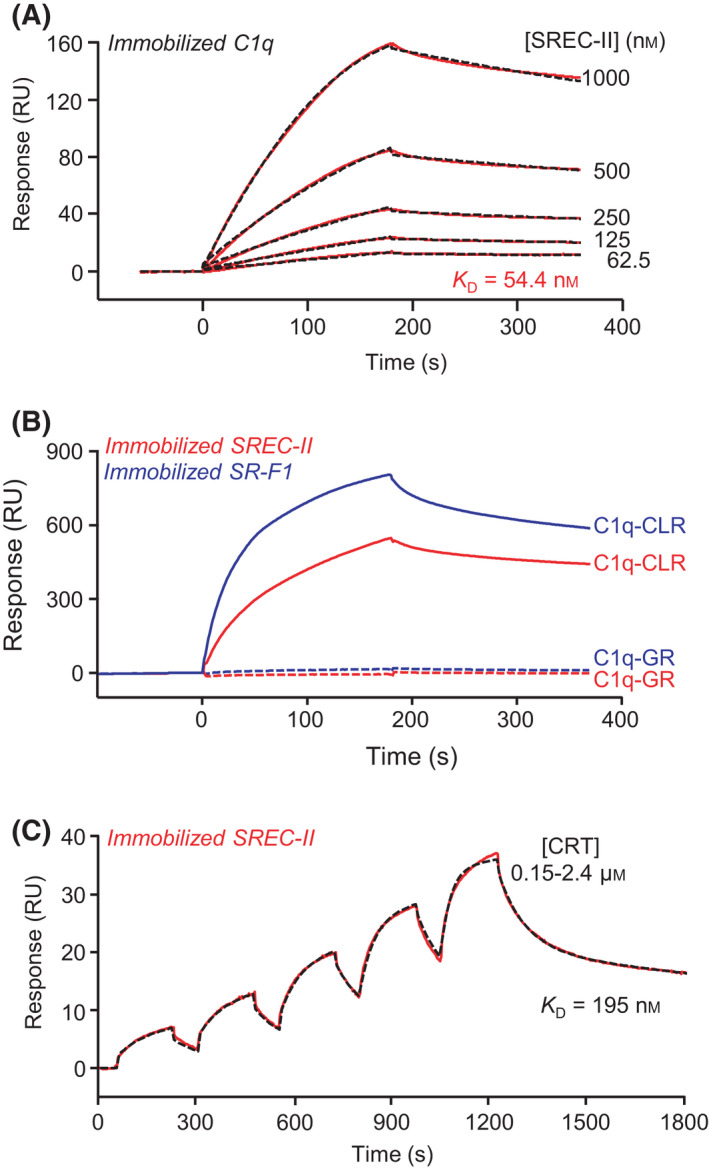
SPR analyses of the interaction of SREC‐II ectodomain with C1q and CRT proteins. (A) SREC‐II(44‐442) was serially diluted and injected over covalently immobilized C1q (18 700 RU) in TBS‐Ca‐P at a flow rate of 20 µL·min^−1^. Fits (shown as dotted lines) and apparent *K*_D_ values were obtained by global fitting of the data using a 1:1 Langmuir binding model. (B) C1q collagen‐like region (C1q‐CLR) and globular region (C1q‐GR) (500 nm) were injected over covalently immobilized SREC‐II(44‐442) (6000 RU) in TBS‐Ca‐P at a flow rate of 20 μL·min^−1^. Immobilized SR‐F1(20‐421) (5500 RU) was used as a control. (C) CRT was serially diluted and injected at five increasing concentrations in single cycle kinetics mode over covalently immobilized SREC‐II(44‐442) (1973 RU) in TBS‐Ca‐P at a flow rate of 30 µL·min^−1^. The fit (shown by a dotted line) and the apparent *K*_D_ value were obtained by global fitting of the data using a two‐state reaction model. The data shown are representative of 4 (A) and 2 (B, C) separate experiments using the T200 or the Biacore 3000 apparatus.

We have demonstrated recently that SR‐F1 ectodomain interacts with complement C1q and CRT [6]. To assess whether SR‐F1 ligands are shared with SREC‐II, SPR interaction analyses were first performed using immobilized C1q (Fig. 3A). SREC‐II bound dose‐dependently to C1q with an apparent *K*_D_ value of 4.5 × 10^−8^ m (Table 2). The affinity of C1q for SREC‐II was about fourfold higher than that determined previously for SR‐F1 (1.9 × 10^−7^ m), mainly because of a higher association rate constant (2.0 × 10^4^ m ^−1^ s^−1^ for SREC‐II and 6.8 × 10^3^ m ^−1^ s^‐1^ for SR‐F1), the dissociation rate constants being of the same order (1.0 × 10^‐3^ and 1.3 × 10^−3^ s^−1^, respectively) (Table 2 and [6]). C1q consists in two functional regions, the collagen‐like regions (CLRs), involved in its association with the C1r and C1s proteases, and the globular regions (GRs), able to recognize various C1q ligands, including immunoglobulins. As shown in Fig. 3B, C1q‐CLRs interacted with immobilized SREC‐II(44‐442), contrary to C1q‐GRs, as observed for SR‐F1(10‐241) (Fig. 3B and [6]).

We next examined the interaction of SREC‐II ectodomain with CRT. As observed previously for SR‐F1 [6], SREC‐II interacted with CRT (Fig. 3C) and the estimated *K*_D_ value (2.9 ± 0.9 × 10^‐7^ m; Table 2) was close to the value previously determined for SR‐F1/CRT interaction (3.3 ± 0.4 × 10^‐7^ m) [6].

#### Interaction of cell surface SREC‐II with maleylated BSA, C1q and calreticulin

**Fig. 4:**
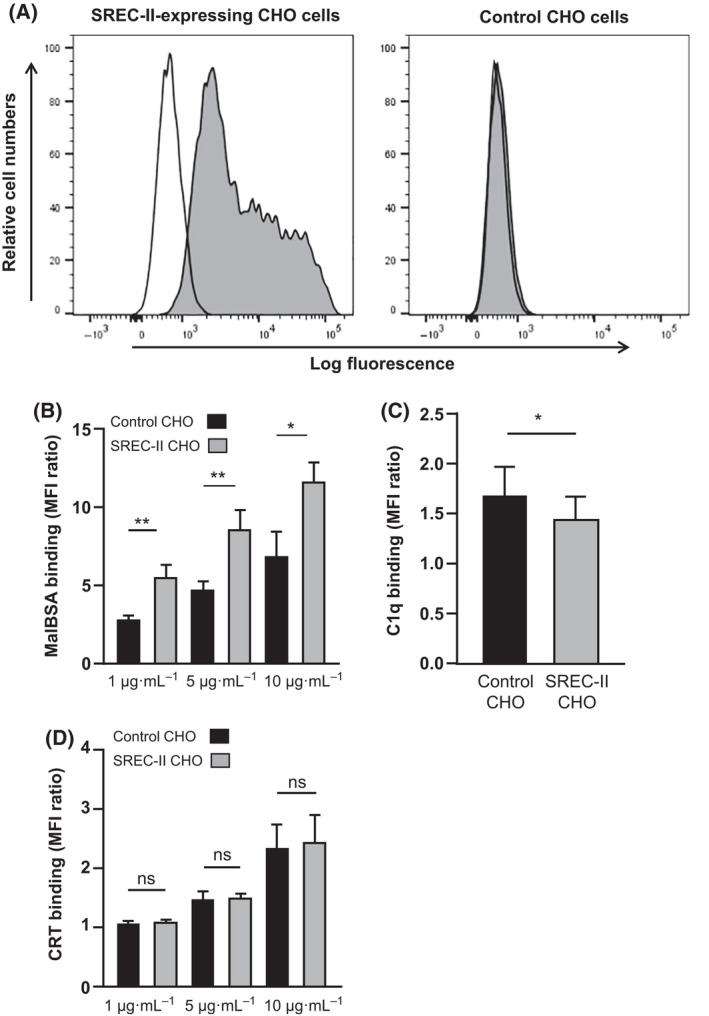
Interaction of cell surface SREC‐II with MalBSA, C1q and CRT. (A) Flow cytometric analysis of SREC‐II expression by transfected CHO cells (left panel); right panel, nontransfected (control) CHO cells. Grey histogram, anti‐SREC‐II Ab; white histogram, isotype control Ab. Results are representative of one out of 5 independent experiments. (B‐D) Binding of MalBSA (B), C1q (80 µg·mL^−1^) (C) and CRT (D) to control and SREC‐II‐overexpressing CHO cells. Experimental conditions are described in Materials and Methods. MFI (median fluorescent intensity) ratio ± SD (*n* = 4). Two‐tailed P value was obtained using t‐test for paired samples after validation by a Shapiro–Wilk normality test. *P < 0.05 and **P < 0.005; ns, not statistically significant.

With a view to exploring SREC‐II interactions in a cellular context, CHO cells were transfected with full‐length SREC‐II cDNA and a stable SREC‐II‐overexpressing CHO cell line was generated as described in Materials and Methods. As shown by flow cytometric analysis, SREC‐II was efficiently exposed at the surface of these cells (Fig. 4A) with 99% SREC‐II‐positive cells and the expression remained stable after one month of culture.

The stable transfectants were incubated with fluorescently labelled MalBSA, and binding was assessed by flow cytometric analysis. Binding of MalBSA to these cells was dose‐dependent and significantly increased by about 40–50% compared with nontransfected CHO cells at the three MalBSA concentrations tested (Fig. 4B). This strongly suggests that SREC‐II is a receptor for MalBSA. Incubation with C1q at a physiological concentration of 80 µg·mL^−1^ did not provide an increase in C1q binding to SREC‐II‐overexpressing CHO cells (*n* = 4) (Fig. 4C) despite the strong interaction demonstrated by SPR (Table 2) between SREC‐II ectodomain and C1q. On the contrary, we measured in all independent experiments an unexpected slightly lower binding (14% decrease) for the stably transfected cells compared with untransfected cells. A similar experiment was performed using CRT, and no specific CRT binding to SREC‐II overexpressing cells could be detected at the three concentrations tested (Fig. 4D). These results indicate that interaction data obtained using isolated purified proteins did not translate directly at the cell surface in the case of SREC‐II and strongly suggest that SREC‐II binding activities depend on other cellular components.

## Discussion

Originally defined as cell surface proteins able to bind chemically modified lipoproteins, scavenger receptors are now recognized as innate immune sensors for a variety of altered self‐ and non‐self‐ligands, with the capacity to mediate endocytosis and, in some cases, trigger signalling events. The scavenger receptor class F family, characterized by the presence of an extracellular domain composed of EGF and EGF‐like domains, comprised three members in the first standardized nomenclature proposed in 2014 [16], including SREC‐I or SR‐F1 (encoded by *SCARF1* gene), SREC‐II or SR‐F2 (encoded by *SCARF2* gene) and Megf10 or SR‐F3 (encoded by *MEGF10* gene). Due to the lack of scavenging activity described so far for SREC‐II, as opposed to SREC‐I and Megf10, only the latter two were considered as ‘true’ scavenger receptors in the present consensus classification proposed in 2017 [5], named SR‐F1 and SR‐F2, respectively. Of note, the name SCARF2 has also been used for skin calmodulin‐related factor 2, a soluble murine Ca^2+^‐binding protein likely involved in the Ca^2+^‐dependent epidermal differentiation process [17, 18], with no relation with scavenger receptors SREC‐II and SR‐F1. Given the high sequence homology of SREC‐II and SR‐F1 extracellular domains and in the light of our recent study showing the capacity of SR‐F1 to bind complement C1q and CRT [6], we explored the capacity of SREC‐II to interact with these proteins and revisited its capacity to interact with classical scavenger receptor ligands.

First, production of the recombinant SREC‐II ectodomain allowed confirmation of its absence of interaction with AcLDL, a typical ligand of scavenger receptors including SR‐F1. However, another common scavenger receptor ligand, MalBSA, obtained by maleylation of BSA lysine residues, proved to interact strongly and dose‐dependently with both SREC‐II and SR‐F1 ectodomains. Binding of both proteins to immobilized MalBSA did not allow determination of affinity constants since dissociation of the complexes required harsh conditions that damaged the surface. The complexes could be dissociated when formed in the reverse configuration (immobilized ectodomains and soluble MalBSA), but the binding curves could not be fitted using simple models, suggesting a complex interaction likely involving multiple binding sites on the receptors EGF‐like domains and on modified BSA. In addition, we showed that both SREC‐II and SR‐F1 interact with another modified BSA molecule obtained by lysyl acetylation. The fact that SREC‐II interacted with AcBSA and not with AcLDL might seem surprising at first sight. However, LDL consists mainly in apolipoproteins and cholesterol molecules and the acetylated protein contents might not be accessible in AcLDL. This hypothesis is consistent with our observation that SREC‐II was able to bind AcLDL in the presence of surfactant P20, a detergent that had a probable deleterious effect on the lipidic part of AcLDL. SREC‐II binding to MalBSA could also be observed at the cell surface using CHO cells stably transfected with the full‐length receptor. It is therefore likely that SREC‐II is able to recognize endogenous modified proteins that remain to be identified. In addition, whether this receptor would be able to trigger endocytosis and thereby exhibit ‘true’ scavenging activity remains another open question.

Second, we showed that C1q binds to the ectodomain of SREC‐II with high affinity, a property shared with SR‐F1 [6], but also with Megf10/SR‐F2 [19]. The interaction involves the collagen‐like regions of C1q, known to contain the binding sites for the extracellular domains of several other C1q receptors including CR1/CD35 [20, 21], LRP1/CD91 [22, 23] and LAIR‐1/CD305 [24, 25]. However, expressing full‐length SREC‐II at the plasma membrane of CHO cells did not induce an increase in C1q binding, which raises the question of the conditions necessary for the fixation of C1q on SREC‐II in a cellular context. Such discrepancy between molecular and cellular analysis was also reproduced for the CRT binding. Of note, in the case of Megf10/SR‐F2, Iram *et al*. demonstrated C1q binding to HEK293 cells transfected with full‐length Megf10 fused to GFP [19]. We could also observe an increase in C1q binding to HEK293‐F cells transfected with full‐length SR‐F1‐GFP, but not with full‐length SREC‐II‐GFP (data not shown), in line with our observations using the CHO cell line. This is a first indication that the choice of the cell model is not necessarily in question and then suggests that the reason of the discrepancy is more likely related to the activation of the binding potential of SREC‐II. Functions of plasma membrane‐anchored receptor are under control of events such as lateral interactions with other molecules, clustering of the receptor, together with conformational changes that modulate its activity. The fact that both soluble and surface‐bound SREC‐II interact with MalBSA, but that the interaction of SREC‐II with C1q and CRT is observed only with the soluble form of the receptor, suggests involvement of distinct binding sites. The MalBSA binding site is likely not impacted by SREC‐II cell partners, whereas those of C1q and CRT seem to be masked, at least partially, at the cell surface.

A second hypothesis might be that, although cell surface SREC‐II does not directly interact with C1q and CRT, proteolytic release of its soluble ectodomain by receptor shedding could allow interaction with both proteins in the extracellular environment. This argument may be further developed by taking into account the reported interaction of cell surface SR‐F1 and SREC‐II through their extracellular domains and the fact that this association prevents binding of SR‐F1 to modified LDL [1]. Our results show that soluble SREC‐II binds to C1q and CRT with a better affinity than SR‐F1, which suggests that it could act as decoy by diminishing the availability of these two proteins for SR‐F1. Thus, by binding to both SR‐F1 and SR‐F1 ligands, SREC‐II could be a regulator of SR‐F1 activity and impact the induced signalling.

Differences between SR‐F1 and SREC‐II C1q binding activities when overexpressed on cell lines could also be relative to their cellular function. Indeed, it has been also shown that phagocytic cells use SR‐F1 to engulf apoptotic cells via interaction with C1q [7], which is also the case for SR‐F2 on astrocytes [19]. In contrast, SREC‐II was not involved in efferocytosis [7].

Despite the fact that cell surface SREC‐II does not interact with known extracellular class F scavenger ligands such as AcLDL and complement C1q, its physiological importance may be inferred from several studies reporting the association between *SCARF2* gene mutations and an extremely rare disease called van den Ende–Gupta syndrome (VDEGS) [26, 27, 28]. VDEGS is inherited in an autosomal recessive manner and is characterized by distinctive facial dysmorphism and skeletal abnormalities with additional infrequent features including joint laxity and recurrent patellar dislocation [29]. How mutations in *SCARF2* gene lead to VDEGS is unknown, but the likely mechanism involves loss of a function potentially important for the proper development of different organs. Currently described mutations include 1‐, 2‐ and 17‐bp deletions located in exons 4, 8 and 11 resulting in a frameshift causing a premature stop codon (Trp148AlafsTer20, Val443ASpfsTer83 and Gln848ArgfsTer95, respectively)). Two other mutations in exons 2 and 4 result in substitution of highly conserved Cys residues predicted to eliminate the formation of the respective disulfide bridge within EGF‐like repeats (Cys64Arg and Cys258Tyr, respectively) and thus affect the folding and putative ligand binding properties of this module. Interestingly, recessive mutations in the *MEGF10* gene have been reported to be associated with a rare disease called early‐onset myopathy–areflexia–respiratory distress–dysphagia syndrome (EMARDD) [30, 31, 32, 33, 34, 35]. The extracellular domain of Megf10 has 17 EGF‐like domains, and the most commonly reported missense mutations are Cys substitutions in EGF‐like modules (Cys118Arg, Cys326Arg, Cys774Arg and Cys810Tyr), expected to disrupt conserved disulfide bonds. In vitro reproduction of the Cys774Arg mutation caused reduced tyrosine phosphorylation in Megf10 intracellular domain [35, 36] and a defect in apoptotic cell uptake and C1q binding [19]. The lack of identified biological function for SREC‐II has precluded such studies.

Overall, the data presented here reveal that SREC‐II exhibits some features of scavenger receptors, such as the ability to bind maleylated BSA, and shares with the extracellular domains of SR‐F1 and Megf10/SR‐F2 the capacity to interact with complement C1q. However, this study certainly represents only a first step in elucidating the physiological role of this enigmatic receptor, possibly involving yet unknown signalling events mediated by its unusually long cytoplasmic tail.

## Conflict of interest

The authors declare that there is no conflict of interest.

## Author contributions

CWP, NMT, YD, PF and DH conceptualized the study. CWP, LP, PTD and SD performed experiments. CWP, NMT, PF, PTD, LP, YD and DH analysed data. CWP and NMT wrote the original draft of the manuscript. All authors read and approved the final version of the manuscript. NMT and YD contributed to funding acquisition.

## Supporting information

**Supplementary Material** List of primers and synthetic SREC‐II(1‐442) coding DNA.

## Data Availability

The data sets used and/or analysed during this study are available from the corresponding author [nicole.thielens@ibs.fr] upon reasonable request.
