# Supplementary material for: Insights into the ligand binding specificity of SREC‐II (scavenger receptor expressed by endothelial cells)
Source: FEBS Open Bio. 2021 Sep 12;11(10):2693–704. doi: 10.1002/2211-5463.13260 (PMC8487046; doi:10.1002/2211-5463.13260)
Supplement: Supplementary file 1 — Supplementary Material List of primers and synthetic SREC‐II(1‐442) coding DNA. [file FEB4-11-2693-s001.pdf]

Supporting information

List of primers (5'-3' sequence)
SREC-II-EcoRIF : TGGTGGAATTCATGGAGGGTGCAGGTCCACGTG
SREC-II-PacR : TCATTAATTAAGCCTTTGCGCTGATTTGTTTCCAGGTG
T7 : TAATACGACTCACTATAGGG
BGHR: TAGAAGGCACAGTCGAGG

Synthetic SREC-II(1-442) coding DNA (GeneCust)
ATGGAGGGTGCAGGTCCACGTGGTGCAGGTCCTGCACGTCGCCGAGGAGCAGGT
GGTCCTCCTTCACCTCTTCTTCCTAGTTTGCTTCTGCTGTTGCTGCTCTGGATGCTG
CCAGACACCGTAGCACCTCAGGAGTTGAACCCTCGTGGTCGTAACGTGTGCCGAG
CTCCAGGTTCACAAGTGCCAACCTGTTGCGCAGGTTGGAGACAGCAGGGTGACG
AGTGTGGTATCGCAGTGTGCGAGGGTAACTCCACCTGTTCCGAGAACGAGGTGTG
CGTGAGGCCTGGAGAGTGTCGATGTCGACACGGATACTTCGGCGCAAACTGCGA
CACCAAGTGCCCACGACAGTTCTGGGGACCAGACTGTAAGGAGCTCTGCAGCTGC
CACCCACATGGACAGTGCGAGGATGTCACCGGACAATGCACCTGCCACGCTAGA
AGGTGGGGAGCAAGATGCGAGCATGCTTGCCAGTGTCAGCACGGAACCTGTCAC
CCAAGAAGCGGAGCTTGCAGATGCGAGCCAGGATGGTGGGGAGCTCAATGTGCC
TCAGCTTGTTACTGCAGCGCCACCTCCAGATGCGATCCACAGACCGGAGCTTGTC
TCTGCCATGCTGGATGGTGGGGGAGAAGTTGCAACAACCAGTGCGCCTGTAACTC
CAGCCCCTGTGAGCAGCAGAGCGGAAGGTGTCAGTGTAGGGAGAGGACATTCGG
CGCTAGGTGTGACAGGTACTGCCAGTGCTTCCGGGGACGGTGTCATCCTGTCGAT
GGCACTTGTGCTTGCGAACCAGGCTACCGGGGCAAGTATTGCCGCGAACCTTGTC
CTGCTGGGTTTTATGGGCTGGGGTGTCGGCGCCGGTGTGGGCAATGTAAGGGGCA
ACAACCCTGTACAGTCGCCGAAGGGCGGTGTCTGACTTGTGAACCCGGCTGGAAT
GGGACAAAGTGTGATCAGCCCTGTGCCACTGGCTTTTATGGCGAAGGCTGTTCTC
ACCGCTGTCCCCCCTGTCGGGATGGGCATGCCTGTAATCATGTGACAGGGAAATG
CACACGCTGCAATGCCGGCTGGATTGGCGATCGGTGCGAAACAAAATGCTCTAAT
GGCACTTATGGCGAAGACTGCGCCTTTGTTTGCGCCGACTGCGGCTCTGGCCACT
GCGACTTTCAGTCTGGGCGCTGCCTGTGCTCTCCCGGCGTTCACGGGCCCCATTGT
AATGTAACTTGCCCCCCCGGCCTCCATGGCGCCGATTGTGCCCAGGCCTGTAGTT
GCCACGAAGATACTTGCGACCCCGTTACTGGCGCCTGCCACCTGGAAACAAATCA
GCGCAAAGGC
